# Characterization and prognosis of estrogen receptor-positive/progesterone receptor-negative male breast cancer: a population-based study

**DOI:** 10.1186/s12957-018-1539-7

**Published:** 2018-12-17

**Authors:** Jin-Li Wei, Jia-Xin Zhang, De-Yuan Fu

**Affiliations:** 1 Department of Thyroid and Breast Surgery, Clinical Medical College of Yangzhou University and Northern Jiangsu People’s Hospital, Yangzhou, China; 2 Department of Breast Surgery, Fudan University Shanghai Cancer Center, Shanghai, China

**Keywords:** Male breast cancer, Progesterone receptor, Estrogen receptor, Cancer-specific survival, Overall survival

## Abstract

**Background:**

The aim of this study was to explore the characteristics and prognostic information of estrogen receptor-positive/progesterone receptor-negative (ER+/PR−) male breast cancer.

**Methods:**

Using the US National Cancer Institute’s Surveillance, Epidemiology, and End Results database, we compared the demographics, clinical characteristics, and outcome of estrogen receptor-positive/progesterone receptor-positive (ER+/PR+) patients with ER+/PR− male breast cancer patients from 1990 to 2010. Two thousand three hundred twenty-two patients with ER+/PR+ tumors and 355 patients with ER+/PR− tumors were included in our study.

**Results:**

ER+/PR− patients were younger (*P* = 0.008) and more likely to be African American (*P* < 0.001) while presented with higher histological grade (*P* < 0.001), larger tumor size (*P* = 0.010), and more invasion to the lymph nodes (*P* = 0.034) and distant sites (*P* < 0.001), thus later stage (*P* = 0.001). Despite higher chance of receiving chemotherapy (51.0% vs 36.5%, *P* < 0.001), ER+/PR− patients experienced significantly worse breast cancer-specific survival (BSCC) (*P* < 0.001) and shorter overall survival (OS) (*P* = 0.003). Multivariate Cox model confirmed that tumor size, lymph node invasion, metastasis, and surgery were independent prognostic factors of both BSCC and OS for ER+/PR− male breast cancer. Age at diagnosis and chemotherapy were significantly associated with OS but not with BSCC.

**Conclusion:**

ER+/PR− male breast cancer was more aggressive and experienced shorter survival than ER+/PR+ patients. The prognosis was mainly associated with tumor size, lymph node invasion, metastasis, and surgery.

## Background

Male breast cancer (MBC) is an uncommon disease, accounting for less than 1% of all breast cancer diagnoses in the USA [1]. However, the annual incidence was reported to increase from 1.0 per 100,000/year in the late 1970s to 1.2 per 100,000/year in 2000–2004 [2]. Due to its rarity, the epidemiology, tumor behavior, treatment, and prognosis remain poorly understood. Current knowledge was mainly based on small series of studies, except for the advancement made by the EORTC 10085/TBCRC/BIG/NABCG International Male Breast Cancer Program. The results of part 1, a retrospective joint central study of 1822 MBC patients, and part 2, a 30-month prospective registry of 557 cases, had been partially released lately [3–6]; thus, further analysis and prospective trials are still yet to be conducted.

Suffering from lack of clinical trials and knowledge on molecular biology, clinicians have to extrapolate treatment strategies for MBC from female breast cancer (FBC) data, despite differences at the protein, genetic, and epigenetic level [7–9]. Although several recent studies have assessed the prognostic factors of MBC, the conclusions are controversial and often blighted by the small number of patients [10–12].

Testing for estrogen receptor (ER) and progesterone receptor (PR) markers has been recommended for all newly diagnosed breast cancer patients by the College of American Pathologists and American Society of Clinical Oncology [13]. Several studies have demonstrated high rates of ER positivity in MBC, for example, Cardoso et al. reported that up to 99.3% of tumors were ER-positive [3, 14, 15]. The range of PR expression is wider than ER among different published reports, from 58.8 to 96% [16]. In FBC, if human epidermal growth factor receptor 2 (HER-2) was negative, ER+/PR− and ER+/PR+ breast cancer would be categorized as luminal B subtype and luminal A subtype, respectively, with different prognosis. Given the fact that HER-2 was dominantly negative in MBC [3, 5, 17], we conducted this population-based study to compare ER+/PR− MBC with ER+/PR+ MBC and further investigate the clinical characterization and prognostic factors of ER+/PR− MBC.

## Materials and methods

Data were obtained from the US National Cancer Institute’s Surveillance, Epidemiology, and End Results (SEER) database [18]. We selected patients diagnosed with breast cancer between 1990 and 2010 according to the following criteria: male, pathological diagnosis of invasive carcinoma, unilateral, ER-positive, and breast as the only primary site. Patients with unknown PR status were excluded. Data extraction was performed by SEER*Stat software version 8.3.2 based on the November 2015 data submission [19]. Marital status was divided into three categories: not married, married, and unknown, with the first one consisting of divorced, separated, single (never married), and widowed. The outcome of interests were breast cancer-specific survival (BCSS) and overall survival (OS). The former was calculated from the date of diagnosis to the date of breast cancer death, and OS was defined as the interval from diagnosis to the death from any cause.

Our study was approved by the ethics committee of our hospital, namely Northern Jiangsu People’s Hospital Ethics Committee. No informed patient consent was needed.

### Statistical analyses

Patient characteristics were compared between ER+/PR+ and ER+/PR− subtypes using a chi-square test or Fisher’s exact test as appropriate. The Kaplan-Meier method was used to construct survival curves. The multivariate Cox regression models were built to assess the independent association of all the variables with BCSS and OS in the ER+/PR+ and ER+/PR− cohorts (forward: LR). Stage, which was defined by tumor size, lymph node invasion, and distant metastasis, was excluded from the model to avoid interference among the variables. Hazard ratios (HR) and their 95% confidence intervals (95% CI) were estimated using the Cox models. Statistical analyses were performed using SPSS 22.0 (Chicago, IL, USA). Two-sided *P* < 0.05 was considered statistically significant.

## Results

### Patient characteristics

A total of 2677 male patients with ER+ invasive carcinoma were included in this study. Two thousand three hundred twenty-two patients had ER+/PR+ tumors, and 355 patients had ER+/PR− tumors. Table 1 shows the demographic and clinical characteristics of patients according to PR status. Compared with PR-positive patients, PR-negative patients were younger (*P* = 0.008), more likely to be African American (*P* < 0.001). PR-negative tumors tended to present with higher grade (*P* < 0.001), larger tumor size (*P* = 0.010), and more invasion to the lymph nodes (*P* = 0.034) and distant sites (*P* < 0.001), thus later stage (*P* = 0.001). Fifty-one percent of PR-negative patients received chemotherapy, significantly higher than PR-positive patients (*P* = 0.001). There was no significant difference between ER+/PR+ and ER+/PR− MBC patients in terms of laterality, marital status, surgery, and radiation therapy (*P* = 0.910, 0.331, 0.623, and 0.089, respectively).

**Table 1:** Demographic and clinical characteristics

Characteristics	PR-positive		PR-negative		*P* value
	*N*	%	*N*	%	
Age at diagnosis					0.008
≤ 40	62	2.7	18	5.1	
41–55	501	21.6	89	25.1	
56–70	901	38.8	145	40.8	
71–85	722	31.1	89	25.1	
> 85	136	5.9	14	3.9	
Race					< 0.001
White	1901	81.9	273	76.9	
Black	266	11.5	70	19.7	
Other	144	6.2	11	3.1	
Unknown	11	0.5	1	0.3	
Laterality					0.910
Right	1211	52.2	184	51.8	
Left	1111	47.8	171	48.2	
Marital status					0.331
Married	1562	67.3	235	66.2	
Not married	662	28.5	110	31.0	
Unknown	98	4.2	10	2.8	
Grade					< 0.001
I	264	11.4	36	10.1	
II	1137	49.0	135	38.0	
III/IV	744	32.0	159	44.8	
Unknown	177	7.6	25	7.0	
Tumor size					0.010
T1	1110	47.8	146	41.1	
T2	906	39.0	149	42.0	
T3	96	4.1	21	5.9	
T4	145	6.2	34	9.6	
TX	65	2.8	5	1.4	
Nodal status					0.034
N0	1175	50.6	153	43.1	
N1	678	29.2	114	32.1	
N2	219	9.4	44	12.4	
N3	152	6.5	32	9.0	
NX	98	4.2	12	3.4	
Metastasis					< 0.001
M0	2172	93.5	311	87.6	
M1	150	6.5	44	12.4	
Stage					< 0.001
I	701	30.2	85	23.9	
II	968	41.7	137	38.6	
III	425	18.3	84	23.7	
IV	150	6.5	44	12.4	
Unknown	78	3.4	5	1.4	
Surgery					0.623
Done	2217	95.5	335	94.4	
Not	98	4.2	19	5.4	
Unknown	7	0.3	1	0.3	
Radiation					0.089
Done	613	26.4	109	30.7	
Not	1709	73.6	246	69.3	
Chemotherapy					< 0.001
Yes	848	36.5	181	51.0	
No/unknown	1474	63.5	174	49.0	

### Survival analysis

**Fig. 1:**
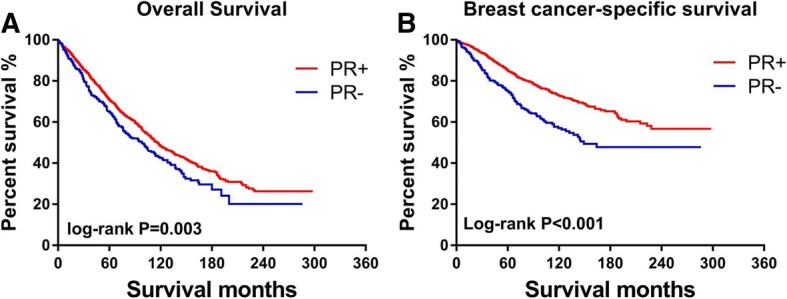
Kaplan-Meier plots of the **a** overall survival and **b** breast cancer-specific survival according to PR status

After a median follow-up of 82 months, 1313 deaths were reported among patients in this study, 625 of which were due to breast cancer. Compared with PR-positive patients, patients with PR-negative breast cancer experienced significantly worse BCSS (*P* < 0.001) and shorter OS (*P* = 0.003) (see Fig. 1).

In the PR-positive MBC cohort, laterality and radiation did not make it into the final Cox model (forward: LR) in the analysis of OS and BCSS. Race, age at diagnosis, marital status, histological grade, tumor size, lymph node status, metastasis, and surgery all exhibited independent prognostic significance. Chemotherapy could significantly improve OS (HR = 1.261, 95% CI 1.088–1.461, *P* = 0.002) but not BSCC, as shown in Table 2.

**Table 2:** Cox proportional hazards regression model multivariate analysis of the overall survival and breast cancer-specific survival in PR-positive cohort (forward: LR)

Variables	OS		BCSS	
	HR (95% CI)	*P* value	HR (95% CI)	*P* value
Age at diagnosis				
≤ 40	Reference		Reference	
41–55	1.143 (0.729–1.791)	0.56	1.245 (0.761–2.035)	0.383
56–70	1.633 (1.056–2.525)	0.027	1.245 (0.768–2.017)	0.374
71–85	3.695 (2.391–5.709)	< 0.001	1.693 (1.032–2.776)	0.037
> 85	7.501 (4.678–12.029)	< 0.001	2.167 (1.123–4.182)	0.021
Race				
White	Reference		Reference	
Black	1.290 (1.069–1.557)	0.008	1.453 (1.129–1.869)	0.004
Other	0.874 (0.662–1.156)	0.346	0.959 (0.628–1.466)	0.848
Unknown	0.862 (0.348–2.138)	0.749	0.000 (0.000–8.644E+62)	0.905
Marital status				
Married	Reference		Reference	
Not married	1.589 (1.397–1.808)	< 0.001	1.508 (1.243–1.829)	< 0.001
Unknown	1.318 (0.957–1.814)	0.08	1.156 (0.690–1.938)	0.582
Grade				
I	Reference		Reference	
II	1.250 (0.996–1.568)	0.054	2.136 (1.297–3.519)	0.003
III/IV	1.470 (1.162–1.858)	0.001	2.775 (1.675–4.596)	< 0.001
Unknown	1.175 (0.875–1.578)	0.283	2.493 (1.412–4.401)	0.002
Tumor size				
T1	Reference		Reference	
T2	1.565 (1.450–1.911)	< 0.001	1.953 (1.567–2.435)	< 0.001
T3	1.858 (1.395–2.475)	< 0.001	1.746 (1.161–2.626)	0.007
T4	1.890 (1.490–2.397)	< 0.001	2.636 (1.863–3.728)	< 0.001
TX	0.873 (0.588–1.296)	0.501	0.981 (0.549–1.753)	0.948
Nodal status				
N0	Reference		Reference	
N1	1.655 (1.426–1.922)	< 0.001	2.317 (1.829–2.935)	< 0.001
N2	1.931 (1.558–2.395)	< 0.001	2.713 (2.002–3.677)	< 0.001
N3	2.362 (1.879–2.968)	< 0.001	4.075 (3.022–5.495)	< 0.001
NX	2.348 (1.741–3.168)	< 0.001	2.194 (1.355–3.553)	0.001
Metastasis				
M0	Reference		Reference	
M1	2.952 (2.348–3.713)	< 0.001	5.412 (4.075–7.189)	< 0.001
Surgery				
Done	Reference		Reference	
Not	2.350 (1.766–3.127)	< 0.001	2.623 (1.830–3.758)	< 0.001
Unknown	1.580 (0.690–3.617)	0.279	1.692 (0.646–4.428)	0.284
Chemotherapy				
Yes	Reference		–	–
No/unknown	1.261 (1.088–1.461)	0.002	–	–

In the PR-negative MBC cohort, laterality, radiation, race, marital status, and histological grade were not included in the final Cox model (forward: LR) in the analysis of OS and BCSS. Tumor size, lymph node invasion, metastasis, and surgery were independent prognostic factors. Age at diagnosis and chemotherapy were significantly associated with OS but not with BCSS. Chemotherapy could reduce the risk of dying from all causes (HR = 1.492, 95% CI 1.073–2.076, *P* = 0.017), as shown in Table 3.

**Table 3:** Cox proportional hazards regression model multivariate analysis of the overall survival and breast cancer-specific survival in PR-negative cohort (forward: LR)

Variables	OS		BCSS	
	HR (95% CI)	*P* value	HR (95% CI)	*P* value
Age at diagnosis				
≤ 40	Reference		–	–
41–55	0.795 (0.365–1.733)	0.564	–	–
56–70	0.812 (0.387–1.705)	0.582	–	–
71–85	1.485 (0.695–3.173)	0.308	–	–
> 85	2.834 (1.087–7.389)	0.033	–	–
Tumor size				
T1	Reference		Reference	
T2	2.047 (1.443–2.902)	< 0.001	2.177 (1.379–3.436)	0.001
T3	5.696 (3.107–10.444)	< 0.001	5.507 (2.749–11.034)	< 0.001
T4	2.565 (1.567–4.201)	< 0.001	3.306 (1.825–5.989)	< 0.001
TX	0.536 (0.143–2.013)	0.356	1.296 (0.365–4.598)	0.688
Nodal status				
N0	Reference		Reference	
N1	1.244 (0.864–1.791)	0.241	1.382 (0.869–2.199)	0.172
N2	2.415 (1.544–3.779)	< 0.001	2.379 (1.381–4.098)	0.002
N3	2.264 (1.605–4.290)	< 0.001	3.509 (1.993–6.181)	< 0.001
NX	2.954 (1.212–7.201)	0.017	3.073 (1.163–8.120)	0.024
Metastasis				
M0	Reference		Reference	
M1	2.311 (1.452–3.677)	< 0.001	3.200 (1.902–5.383)	< 0.001
Surgery				
Done	Reference		Reference	
Not	2.143 (1.130–4.064)	0.020	3.120 (1.621–6.006)	0.001
Unknown	2.629 (0.336–20.549)	0.357	1.683 (0.215–13.191)	0.62
Chemotherapy				
Yes	Reference		–	–
No/unknown	1.492 (1.073–2.076)	0.017	–	–

**Fig. 2:**
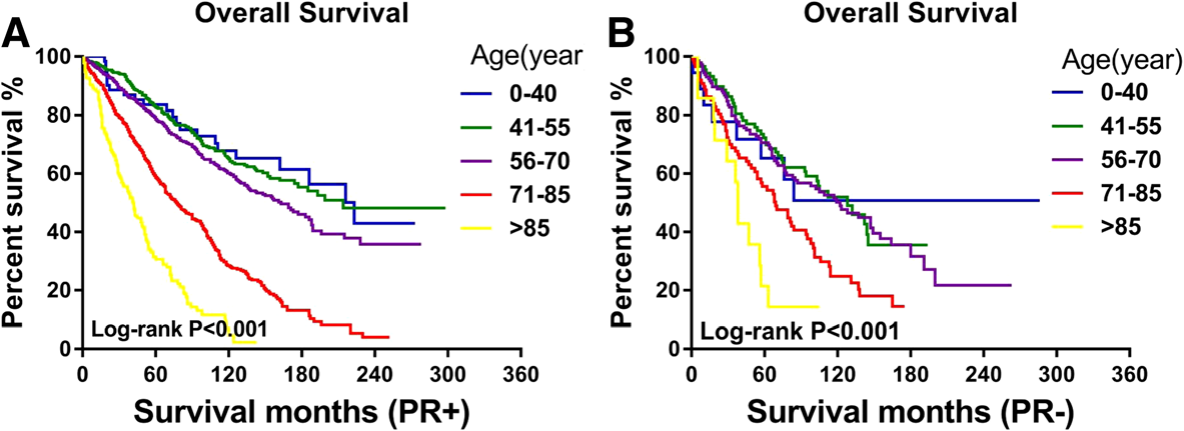
Kaplan-Meier plots of the overall survival according to age groups within **a** PR-positive and **b** PR-negative cohorts

As age at diagnosis was related to OS in both the PR-positive and PR-negative patients, we constructed the survival curves and conducted a pair-wise comparison among different age groups to further explore the difference between PR-positive and PR-negative MBC, as shown in Fig. 2 and Table 4. For PR-positive MBC, the OS of patients younger than 40 was not significantly different from patients aged 41 to 55 (*P* = 0.800) and 56 to 70 (*P* = 0.154) but better than patients aged 71 to 85 (*P* < 0.001) and older (*P* < 0.001). The OS of patients aged 41 to 55 was significantly better than the following groups (*P* = 0.010, *P* < 0.001, and *P* < 0.001, respectively). For PR-negative MBC, the OS of patients younger than 40, patients aged 41 to 55, and patients aged 56 to 70 did not change dramatically (*P* = 0.951, 0.772, and 0.738, respectively). Survival declined significantly with age after 70, as shown in Table 4.

**Table 4:** Comparison of the overall survival among different age groups (log-rank)

PR status	Age at diagnosis	≤ 40	41–55	56–70	71–85	> 85	Survival months
		*P* value	*P* value	*P* value	*P* value	*P* value	Mean ± SD
Positive	≤ 40		0.800	0.154	< 0.001	< 0.001	184.0 ± 14.3
	41–55	0.800		0.010	< 0.001	< 0.001	193.7 ± 6.7
	56–70	0.154	0.010		< 0.001	< 0.001	153.3 ± 4.9
	71–85	< 0.001	< 0.001	< 0.001		< 0.001	91.2 ± 3.1
	> 85	< 0.001	< 0.001	< 0.001	< 0.001		48.8 ± 3.3
Negative	≤ 40		0.951	0.772	0.088	0.024	164.7 ± 31.3
	41–55	0.951		0.738	0.001	< 0.001	117.50 ± 8.0
	56–70	0.772	0.738		< 0.001	< 0.001	129.2 ± 9.3
	71–85	0.088	0.001	< 0.001		0.037	78.6 ± 6.4
	> 85	0.024	< 0.001	< 0.001	0.037		44.4 ± 8.1

## Discussion

MBC is substantially different from FBC, arising with increasing frequency due to BRCA2 mutations with differential effects by gender of single nucleotide polymorphisms (SNPs) [20]. The rarity of MBC resulted in difficulty in operating randomized, controlled clinical trials and limited prognostic information and suboptimal treatment. Only 3 out of the 12 breast cancer trials that included male patients are phase 3 clinical trials, and just 1 trial is actively recruiting [5]. The implications for PR positivity have long been a focus of debate. Some researchers recommended the elimination of PR testing from the routine diagnostic work-up of invasive breast cancer [13]. Other researchers advocated assessment of PR status to distinguish subsets of ER-positive and ER-negative tumors [21]. Also, PR status was suggested as a useful tool for selecting initial therapy, because ER+/PR− tumors might benefit more from initial treatment with an aromatase inhibitor [22]. However, tamoxifen is still the standard endocrine therapy in MBC patients [17]. In our study, we found that the clinical characteristics and prognosis of ER+/PR− MBC were different from ER+/PR+ MBC, the former being more aggressive and experiencing a much shorter OS and BCSS. Given the fact that normally endocrine therapy would be administered to both ER+/PR+ MBC and ER+/PR− MBC patients, the survival difference possibly lay more in tumor behavior than treatments. This verified the importance of PR testing. Besides, according to our study, the prognosis of ER+/PR− MBC patients was significantly related to tumor stage and surgery other than demographic factors like marital status and race; herein, early detection, diagnosis, and intervention were of great importance to improve the outcome of these patients.

The median age at diagnosis of MBC is 65–69 years old [3, 6, 23–25] in the West counties and a little bit younger in Asia [26] and the Middle East [27]. Most literature has validated the prognostic role of age at diagnosis [12, 24, 26, 28, 29]. Our study was in agreement with the previous research. Nevertheless, we found different effect of age between the PR-positive and PR-negative MBC. OS dropped significantly for PR-positive patients older than 55 years, while patients younger than 70 years old experienced similar OS in PR-negative group. Furthermore, age was not independently related to BCSS in PR-negative MBC patients. The analysis of EORTC 10085/TBCRC/BIG/NABCG International Male Breast Cancer Program partially supported our results [3]. Compared with patients diagnosed ≤ 40 years old, patients diagnosed ≥ 75 years old experienced a 25% higher mortality risk. Nonetheless, the authors did not conduct strata analysis according to breast cancer subtypes, and only deaths following a distant relapse were considered breast cancer mortality event. We assume that older onset of age with a high presence of comorbidities could explain the divergence between OS and BCSS. 32.6% of MBC patients were diagnosed with comorbidities, including hypertension, diabetes, and ischemic heart disease [26]. Nearly 40% of MBC patients died from causes unrelated to their breast cancer [24].

Histological grade is representative of the “aggressive potential” of the tumor and would be expected to predict the survival of MBC. Some literature did report that tumor grade was a predictor of OS and/or BCSS [25, 28, 30], so did our analysis of the PR-positive cohort. On the contrary, Vermeulen et al. [6] found that tumor grade was not independently associated with survival, so did our analysis of the PR-negative patients and some other research [3, 31]. Different “scoring systems” were applied for determining the grade of a breast cancer, including four-tier grading scheme and three-tier grading scheme, which undermined the comparison among different results. Also, the grading system that was initially developed for FBC may not be suitable in the MBC setting. Last, MBC could be a heterogenous disease with different subtypes exhibiting different prognostic patterns, as our work demonstrated.

Interestingly, chemotherapy was confirmed an independent prognostic factor in the multivariate Cox analyses of OS but did not reach significance with this test in BCSS, neither in ER+/PR+ nor ER+/PR− cohort, as shown in Tables 2 and 3. Since few studies analyzed OS and BCSS of MBC at the same time, our finding was not echoed. There might be some possibilities: first, the drugs somehow reduced the risk of dying from causes other than cancer; second, we did not use HER-2 status in the model, which was not available until 2010, so the conclusion might be partial. The application of the 21-gene breast recurrence score (RS) may shed some light on the option of chemotherapy. After testing 38 MBC patients, Turashvili et al. [32] found similar RS distribution in MBC and FBC patients. Besides, RS testing was declared to play a prognostic role in MBC [7]. Larger studies with different cohorts are needed to further identify the risk factors and optimize treatments for MBC patients.

We acknowledge some limitations to our study. We do not have the information regarding HER-2 status, as mentioned above. Also, as a retrospective analysis, our study may have introduced biases. Despite these limitations, our study, to our best knowledge, was the first to expound the characterizations and prognosis of PR-negative MBC. Also, our SEER-based study included the data on systemic treatments of this population, which was recently updated.

In conclusion, ER+/PR− MBC, compared with ER+/PR+ MBC, presented with more aggressive behavior and poorer survival. The prognosis was independently associated with stage and clinical intervention; thus, early diagnosis and individualized treatment were warranted to improve the outcome.

## Abbreviations

BCSS: Breast cancer-specific survival
CI: Confidence intervals
ER: Estrogen receptor
FBC: Female breast cancer
HER-2: Human epidermal growth factor receptor 2
HR: Hazard ratios
MBC: Male breast cancer
OS: Overall survival
PR: Progesterone receptor
RS: Recurrence score
SNPs: Single nucleotide polymorphisms
